# Metabolic Syndrome and Its Components in Young Adults Conceived by ICSI

**DOI:** 10.1155/2018/8170518

**Published:** 2018-05-03

**Authors:** F. Belva, M. Bonduelle, S. Provyn, R. C. Painter, H. Tournaye, M. Roelants, J. De Schepper

**Affiliations:** ^1^Centre for Medical Genetics, Universitair Ziekenhuis Brussel (UZ Brussel), Laarbeeklaan 101, 1090 Brussels, Belgium; ^2^Department of Anatomical Research and Clinical Studies, Vrije Universiteit Brussel, Laarbeeklaan 103, 1090 Brussels, Belgium; ^3^Department of Obstetrics and Gynaecology, Academic Medical Centre, Meibergdreef 9, 1105 AZ Amsterdam, Netherlands; ^4^Centre for Reproductive Medicine, Universitair Ziekenhuis Brussel (UZ Brussel), Laarbeeklaan 101, 1090 Brussels, Belgium; ^5^Environment and Health/Youth Health Care, Department of Public Health and Primary Care, Kapucijnenvoer 35, 3000 Leuven, Belgium; ^6^Pediatric Endocrinology, Universitair Ziekenhuis Brussel (UZ Brussel), Laarbeeklaan 101, 1090 Brussels, Belgium

## Abstract

**Background:**

Intracytoplasmic sperm injection (ICSI) conception presents the early embryo with a radically different environment, which may lead to permanent alterations to key cardiometabolic processes. Blood pressure, indicators of insulin resistance, and lipid profiles have previously been studied in offspring born after in vitro fertilisation (IVF) and ICSI, with conflicting findings. Also, results in young adults born after ICSI are lacking.

**Aim:**

We investigated if young adult men and women conceived by ICSI more frequently have metabolic syndrome and its individual features in comparison to spontaneously conceived controls.

**Design:**

Cardiometabolic and anthropometric parameters from 126 longitudinally followed young adults conceived by ICSI were compared to those of 133 controls.

**Results:**

At age 18 years, only 1 of the participants displayed the metabolic syndrome (1 control woman). Mean concentrations of total cholesterol, triglycerides, insulin, HOMA-IR, and blood pressure were comparable between the ICSI conceived and control participants. A higher proportion (19.6%) of men conceived by ICSI had low (<40 mg/dl) HDL cholesterol compared to controls (5.6%).

**Conclusions:**

While men conceived by ICSI, but not women, had lower mean HDL cholesterol concentrations in comparison to controls, other markers of the metabolic syndrome were not affected by the mode of conception.

## 1. Introduction

The number of children born after assisted reproductive technology (ART) is rapidly increasing worldwide. Although, to date, more than 5 million children have been conceived through in vitro fertilisation (IVF) and intracytoplasmic sperm injection (ICSI), the offspring's long-term health outcomes of assisted reproductive techniques are poorly known [1]. Notwithstanding this gap in knowledge, potential health risks following IVF are of matter of concern from a public health point of view. Indeed, within the concept of the developmental origins of adult disease, it is now generally accepted that the early embryo's environment can induce changes in development and function with long-lasting consequences in adult life including diabetes and cardiovascular disease [2].

The metabolic syndrome predicts a twofold increase in cardiovascular disease [3], consisting of independent, common risk factors: central obesity, glucose intolerance, insulin resistance, dyslipidemia, and hypertension. The clustering of metabolic risk factors starts already in childhood [4, 5] and tends to persist from childhood into adulthood [6]. The study of the metabolic syndrome is particularly challenging during the transition period to adulthood given the rapid change in body composition and the gender-related developmental changes in insulin resistance [7, 8]. The pathophysiological mechanisms underlying the metabolic syndrome are not fully known, but insulin resistance and visceral obesity are considered as major causes.

Since the introduction of IVF and ICSI in clinical practice, concerns of unfavorable general health in neonates and children after ART were addressed and studies investigating risk factors for future cardiovascular disease and/or diabetes were initiated [9, 10]. Since ART inherently differs from spontaneous conception, the manipulation of the gametes and the embryo culture might affect normal cardiovascular and metabolic function, as has been shown in animals [11–14]. More precisely, ART-conceived mice were shown to have high blood pressure [11, 12] and altered glucose metabolism [13, 14]. Accumulating evidence suggests that ART is associated with epigenetic modifications in genes that are linked to fetal growth and reprogramming which may increase the risk of adverse cardiometabolic outcomes [15–17].

From 2007 onwards, there have been reports of increased blood pressure, dyslipidemia, and higher fasting glucose levels in ART-conceived children [18–21], but some studies found a more favorable lipid profile [9] or similar blood pressure readings [22]. In order to investigate the prevalence of the metabolic syndrome after ICSI in adulthood, we performed a comprehensive investigation of the metabolic syndrome and its components, including abdominal circumference, fasting insulin and glucose levels, blood pressure and plasma LDL, and HDL cholesterol levels in the worldwide eldest cohort of adults conceived by ICSI, currently aged between 18 and 20 years. We hypothesized that the cardiometabolic profile might become less favourable during the transition from adolescence into adulthood as a consequence of the naturally increasing insulin resistance and visceral adiposity, resulting in an increase in outright metabolic syndrome [23].

## 2. Subjects and Methods

### 2.1. Set-Up and Study Groups

This study is part of a larger project investigating cardiometabolic and reproductive health in young adults, both female and male, that were conceived by ICSI. Therefore, several examinations including a physical examination, biometry, semen analysis, and blood sampling were performed. Results of the reproductive profile of adults conceived by ICSI are reported elsewhere [24, 25].

Young adults were eligible for inclusion if they were singleton, Caucasian, and aged between 18 and 22 years during the study period (March 2013 to April 2016).

These ICSI adults were born between 1992 and 1996 and are part of a cohort that has been prospectively followed since birth [26]. Given that our centre has a tradition of monitoring children both for psychological and medical outcome, we focused on those that were singleton, Caucasian, and Dutch-speaking. The main reasons for this selection are (1) the higher incidence of gestational diabetes in non-Caucasians [27], (2) known differences in body composition and growth trajectories [28] which are key outcomes in our follow-up studies, and (3) the psychological assessment in parallel to the medical examination from the age of 5 years onwards, which was limited to Dutch-speaking Caucasians in order to minimise possible sociocultural differences. The ICSI technique was first described in our university in the early 90s. Since its introduction in clinical practice, all children conceived after ART in our centre are prospectively followed since birth. Follow-up studies are performed in a standardised way ever since. Briefly, as soon as a follow-up questionnaire from a gynaecologist/paediatrician mentioning a live-born after ART has reached us, a dedicated study nurse invites the parents by phone to come to our centre for a medical examination at the age of 2 months. At that time, data from the questionnaires are double-checked and further completed with information from the parents when needed. In case of possible medical problems, the paediatrician is approached. Follow-up at later ages is conducted in the same way. Furthermore, clinical assessments are scheduled at the age of 1 year, 2 years, 5 years, 8 years, and 14 years.

All parents of eligible young ICSI adults in our database (*n* = 423) were sent a letter explaining the background and set-up of the study. Shortly after, these parents were contacted by phone in order to explore their own and their children's willingness to participate. After consent of the parents, the young adults were approached and invited to participate. They were not approached directly, since we learned from our study conducted at 14 years of age that 20% of the parents did not disclose the mode of assisted conception to their child.

From the 423 eligible ICSI families (215 with a male offspring and 208 with a female offspring), 303 (149 male and 154 female) could be reached.

Because some parents decided that “the family” was not interested to participate and some had not disclosed the mode of conception to their child, we only obtained permission from 237 parents to approach their children. Hence, we could effectively invite 119 men and 118 women, of whom 56 (47.0%) men and 71 (60.2%) women agreed to participate.

A control group of 138 spontaneously conceived peers (57 men and 81 women) aged between 18 and 22 years was recruited at college and university campuses by oral and written campaigns. Only young adults born after spontaneous conception without the use of any hormonal stimulation were eligible as controls. Hence, the controls were cross-sectionally recruited; none of the controls had participated in earlier studies.

For the present analysis, the results from 4 control men, 1 ICSI woman, and 1 control woman who combined higher education with a professional sports career were omitted, since these subject are not indicative for men and women of the general population. The average number of hours of sports per week was >10 hours for the 4 excluded men born after spontaneous conception, 12 hours for the woman born after ICSI, and 12 hours for the woman born after spontaneous conception. Therefore, the final study population consists of 56 ICSI and 53 control men and 70 ICSI and 80 control women.

### 2.2. Measurements

#### 2.2.1. Anthropometric Measurements

Body weight was measured using a class III flat scale (SECA 877; Hamburg, Germany), and the standing height was measured to the nearest 1 mm using a stadiometer (SECA 217; Hamburg, Germany). Body mass index (BMI) was calculated as weight in kilograms divided by the square of height in meters (kg/m^2^). Measurement of the waist was performed with a flexible steel tape (Lufkin W606PM; Maryland, USA), halfway the distance between the lower costal margin and upper end of the crista iliaca.

All anthropometric measurements were taken in duplo. If the difference between the first and the second measurement was ≥1% for the circumferences, a third measurement was performed. The final result was the average between the two measurements that were closest to each other.

The body roundness index (BRI), a marker that reflects the cardiovascular health status of an individual, was additionally calculated [29] according to the formula.

(1) $$\begin{array}{c} BRI=364.2-365.5\times \sqrt{1-\left(\frac{{\left(WC/2\pi \right)}^{2}}{{\left(height/2\right)}^{2}}\right)}. \end{array}$$

Smaller values indicate leaner individuals and larger values are associated with rounder individuals.

#### 2.2.2. Blood Pressure Readings

We measured blood pressure twice using an automated electronic device (Spot Vital Signs, Welch Allyn, Inc., USA) on the nondominant arm when the subject had been seated for at least 5 min. The device has passed international validation protocols for accuracy [30, 31]. Resting systolic and diastolic blood pressure was calculated as the mean of the two measurements.

#### 2.2.3. Blood Sampling

Fasting blood samples were collected between 9 and 11 a.m. Plasma glucose, cholesterol, HDL cholesterol, and triglycerides were analysed using Vitro 4000 (Ortho Clinical Diagnostics). The concentration of LDL cholesterol was calculated. Insulin levels were measured using the Elecsys Insulin kit in combination with the Cobas 411 (Roche Diagnostics, Germany) equipment. The homeostasis model assessment-estimated insulin resistance (HOMA-IR) index was calculated according to the original formula [32].

#### 2.2.4. Questionnaires

All participants were asked to complete a standardised questionnaire on paper covering a large series of health parameters about themselves and their family, including information on several lifestyle factors (sports, smoking habits, alcohol consumption, and recreational drugs), medication intake, and in utero exposure to tobacco during pregnancy. Since all participants were clinically assessed, information was collected from all participants, even in the absence of a completed questionnaire. In case of missing data, the participants were contacted again for further clarification.

### 2.3. Definition of Metabolic Syndrome

Several definitions of metabolic syndrome are currently in use. We chose to use the most broadly applied definition, with the strongest association with later cardiometabolic disease: the revised National Cholesterol Education Program Adult Treatment Panel III (NCEP ATP III) [33]. According to the NCEP ATP III definition, metabolic syndrome is present if three or more of the following five criteria are met: waist circumference over 102 cm (men) or 88 cm (women), blood pressure over 130/85 mmHg, fasting triglyceride level over 150 mg/dl, fasting HDL cholesterol level less than 40 mg/dl (men) or 50 mg/dl (women), and fasting blood sugar over 100 mg/dl.

### 2.4. Informed Consent and Ethics Committee

The present study is part of a larger project on reproductive and cardiometabolic health in young adults conceived by ICSI, which was approved by the Ethics Committee of the Universitair Ziekenhuis Brussel (UZ Brussel). Written informed consent was obtained from all ICSI-conceived men and women and of all spontaneously conceived men and women participating in the current study.

### 2.5. Statistical Analysis

Descriptive statistics were calculated for all parameters of the sample. Mean and standard deviations are reported. Plasma triglycerides, serum insulin, and HOMA-IR were logarithmically transformed to normalize their distribution. For ease of interpretability, reported means and 95% confidence intervals were back-transformed. Cholesterol and glucose were distributed close to normal and were analysed without transformation. A chi-square test (Fisher's exact test for small numbers) was used to compare categorical variables in ICSI and control subjects, and a *t*-test to compare (log-transformed) continuous variables. Data analysis was performed using SPSS software version 23.

Multiple linear regression analysis was used to investigate differences in metabolic syndrome and its individual features between the two study groups. Results are expressed as unstandardized regression coefficients (B) with a 95% confidence interval (95% CI). Covariates known to affect metabolic parameters, and covariates that differed among groups, were included in the final model: age, alcohol consumption > 5 units/week (yes/no), hours of sport per week (continuous), current BMI mother (continuous), and current BMI father (continuous). For the analysis of insulin and HOMA-IR, standing height and birth weight as well as gestational age were included in the models as additional covariates.

The sample size of the project was determined by a power analysis based on the total sperm count since the reproductive health of young adults conceived by ICSI was the primary outcome of this project. In the present analysis of 56 ICSI and 53 control men and 70 ICSI and 80 control women, differences exceeding approximately 0.4 times the standard deviation in men and 0.32 times the standard deviation in women are statistically significant with an alpha level of less than 5% (two-sided test). For example, the minimum detectable difference in blood pressure between cases and controls is approximately 4 mmHg systolic or 3.5 mmHg diastolic blood pressure. Also, the present sample sizes allow us to detect at least one case of the metabolic syndrome in the ICSI or control groups with a probability of 80% when the prevalence exceeds 3% in men and 2% in women.

## 3. Results

### 3.1. Characteristics of the Study Population

Men conceived by ICSI were slightly younger than control men. Alcohol consumption was more prevalent in men conceived by ICSI (Table 1).

Women conceived by ICSI engaged in fewer hours of sports per week, while smoking habits and alcohol consumption were comparable to control women (Table 1).

### 3.2. Anthropometric Measurements

In both men and women, body weight, height, waist circumference, and body roundness index were comparable between the two groups (Table 1), even after adjustment for covariates (Table 2).

### 3.3. Blood Pressure

Mean systolic and diastolic blood pressure was comparable in the ICSI and the control group in men and in women (Table 1). Adjustment for confounders did not change the results (Table 2).

### 3.4. Blood Analytes

In men, mean concentrations of fasting glucose and cholesterol and back-transformed mean concentrations of triglycerides, insulin, and HOMA-IR were comparable between the two groups, even after controlling for covariates (results not shown). However, mean HDL cholesterol was lower in men conceived by ICSI in comparison with control men (Table 3). This difference remained significant after controlling for the studied covariates (B: −5.3; 95% CI: −10.0, 0.4; *P* = 0.03) (results not shown).

In women, fasting cholesterol, HDL cholesterol, LDL cholesterol, triglycerides, insulin, and HOMA-IR were comparable between the two groups (Table 3). Women conceived by ICSI had a lower mean fasting glucose level than control women, but this difference was no longer statistically significant after controlling for covariates (B: −2.3; 95% CI −4.8, 0.1; *P* = 0.06) (results not shown).

### 3.5. Metabolic Syndrome

No men or women conceived by ICSI and one spontaneously conceived woman had the metabolic syndrome, defined as having 3 or more components. While the absence of cases does not allow us to provide an estimate of the prevalence in these groups, the upper boundary of a 95% confidence interval is approximately 6% in ICSI and control men and approximately 5% in women. The number of participants with individual features of the metabolic syndrome is shown in Table 4. Only 2 men conceived by ICSI and 2 control men had a combination of two risk factors. Three women conceived by ICSI and 3 control women had a combination of two risk factors.

Men conceived by ICSI had a significantly higher prevalence of decreased (<40 mg/dl) HDL cholesterol levels (19.6% versus 5.6%; *P* = 0.02).

## 4. Discussion

We report for the first time the results of a comprehensive investigation of an ICSI-conceived cohort of 56 young adult men and 70 young adult women, with regard to the metabolic syndrome and its components, including abdominal adiposity, blood pressure, lipid levels, fasting glucose, and insulin resistance.

Up to now, parameters of the metabolic syndrome have only been studied in a small group of 14 young adults conceived after IVF but not ICSI [34]. In our study, we found that men but not women conceived by ICSI presented with a 3 times higher prevalence of an HDL cholesterol concentration below 40 mg/dl than controls. Other components of the metabolic syndrome were not more prevalent among ICSI-conceived adults compared to peers born after spontaneous conception. Furthermore, fasting insulin and HOMA-IR were not higher among men and women conceived by ICSI than among controls.

Standard anthropometric evaluation showed a comparable adult standing height between young adults conceived by ICSI and spontaneously conceived peers, confirming our previous findings that not only childhood growth but also the pubertal growth phase in ICSI offspring is comparable to the reference population [35]. This is important because standing height in adults has been shown to relate to insulin secretion, type 2 diabetes risk, and cardiovascular disease [36]. Waist circumference in women conceived by ICSI was not different from controls, but a trend to a higher waist circumference in men conceived by ICSI remained after adjusting for confounders. Previous studies found that abdominal adiposity, as assessed by waist circumference, was normal in ART-conceived preschool children [21, 37] and adolescents [38]. However, we previously reported that 14-year-old ICSI female adolescents but not male adolescents had a more central fat distribution pattern, as assessed by waist circumference and sum of subscapular and suprailiacal skinfolds, in comparison with spontaneously conceived adolescents [39]. We speculate that differences in recruitment strategies of controls and differences in covariate selection as well as physiological changes in visceral fat that occur after menarche might explain the differences between our consecutive anthropometric studies in this cohort [21, 22].

Lower HDL cholesterol levels in men conceived by ICSI was the main findings of our blood analysis. Most studies in ART offspring have found comparable HDL cholesterol concentrations during childhood and adolescents [20, 40, 41], with the exception of the study by Sakka et al. [19] reporting higher HDL concentrations in IVF offspring. In our study, the lower sport involvement in men conceived by ICSI might be a factor that contributed to the lower HDL cholesterol concentration. We also speculate that the natural increase in insulin resistance at the end of adolescence in men might also have been a relevant factor for the lower HDL values. Chen et al. found a decreased peripheral insulin sensitivity in a small group of 14 young adults and a control group of 20 adults, matched for age, BMI, smoking, alcohol consumption, and general health, despite similar fasting glucose and insulin concentrations [34]. In accordance with their findings and the presence of normal fasting insulin concentration among IVF-ICSI offspring in most studies performed during childhood [9, 18, 19, 41], we did not find a difference between ICSI young adults and their spontaneously conceived peers in our study. To rule out some degree of insulin resistance, clamp studies are recommended in future research.

Another reassuring finding of our study was that blood pressure was comparable among the study groups, both for men and women. A recently published meta-analysis including 10 studies showed a small but statistically significant difference in blood pressure of IVF-ICSI offspring and naturally conceived controls [42]. However, the authors of this meta-analysis clearly pointed to the fact that the pooled effect size was small and the results mainly relied on studies with positive effect estimates and therefore should be interpreted with caution.

The presented results in young adulthood should however be interpreted with caution. The sample size of our cohort was modest but we were able to account for major confounding factors in the analysis of the different components of the metabolic syndrome. Despite our efforts, we cannot rule out that the control group we recruited may not be entirely representative of the general population in terms of cardiometabolic health. However, in terms of reproductive health parameters, we found no evidence for selection bias [25, 43]. Furthermore, vascular functioning (vascular wall thickness and cardiac function) was not included in our study but has been reported to be suboptimal in ART offspring in several studies [44–47]. Therefore, cardiovascular morphology and function measurements as well as 24 h ambulatory blood pressure measurements should be performed in addition to standard blood pressure measurements in ART offspring. Furthermore, the endothelial function was not investigated. The long-term cardiovascular effect of small changes in metabolic risk parameters, especially in those with a lower level of physical activity, needs further attention in the coming decades.

## 5. Conclusion

We found no evidence for an increased cardiometabolic risk in young adulthood after ICSI conception, with the exception of lower HDL cholesterol concentration in men. This finding needs confirmation from further follow-up studies in middle-aged men and women. Also, additional measurements of vascular functioning and insulin resistance would provide more detailed information on cardiometabolic morbidity in ICSI-conceived individuals.

## Figures and Tables

**Table 1:** Characteristics^∗^ of ICSI-conceived and spontaneously conceived control men and women.

	Men			Women		
	ICSI *N* = 56	Control *N* = 53	*P* value	ICSI *N* = 70	Control *N* = 80	*P* value
Birth weight (g)	3371 (596)	3482 (389)	0.29	3150 (432)	3182 (521)	0.72
Age (years)	19.4 (0.7)	20.0 (1.2)	0.001	19.3 (0.6)	19.5 (0.9)	0.10
Weight (kg)	74.9 (9.9)	73.1 (10.0)	0.35	60.2 (8.5)	62.1 (8.8)	0.16
Height (cm)	180.4 (6.8)	179.8 (6.2)	0.66	165.4 (19.0)	167.1 (6.2)	0.40
BMI (kg/m^2^)	23.0 (2.7)	22.6 (2.8)	0.45	21.5 (3.2)	22.2 (2.6)	0.14
Smoking (yes/no)	12 (24.0%)	6 (11.5%)	0.10	3 (4.3%)	6 (7.6%)	0.50
Alcohol consumption (yes/no)	42 (80.7%)	31 (64.6%)	0.76	47 (68.1%)	56 (72.7%)	0.58
>5 units/week	26 (46.4%)	15 (28.3%)	0.07	7 (10.1%)	8 (10.4%)	0.99
Sports (yes/no)	38 (71.8%)	43 (87.8%)	0.05	44 (63.8%)	62 (78.5%)	0.07
Sports (hours/week)	4 (4)	5 (3)	0.28	2 (2)	3 (3)	0.001
Hormonal contraceptive use (yes/no)				59 (83.1%)	59 (72.8%)	0.13
Waist circumference (cm)	80.0 (7.4)	77.3 (6.9)	0.10	70.9 (6.1)	70.2 (6.0)	0.53
Body roundness index	2.4 (0.8)	2.2 (0.68)	0.25	2.1 (0.7)	2.0 (0.6)	0.60
Blood pressure						
Systolic (mmHg)	121 (9)	123 (8)	0.17	116 (9)	118 (8)	0.26
Diastolic (mmHg)	70 (8)	71 (7)	0.55	73 (7)	74 (7)	0.66

^∗^Mean (SD) or *n* (%); *P* values when comparing ICSI and control offspring with a *t*-test or chi-squared test.

**Table 2:** Linear regression analysis of cardiometabolic risk factors in ICSI-conceived and control men and women: unadjusted and adjusted for covariates.

	Unadjusted			Adjusted for covariates^∗^		
	B^∗∗^	95% CI	*P* value	B^∗∗^	95% CI	*P* value
*Men*						
BMI (kg/m^2^)	0.4	−0.6; 1.5	0.45	0.6	−0.5; 1.6	0.29
Waist circumference (cm)	2.7	−0.5; 5.9	0.10	3.9	0.0; 7.8	0.05
Body roundness index	0.2	−0.1; 0.5	0.25	0.2	−0.1; 0.6	0.25
Systolic blood pressure (mmHg)	−2.3	−5.7; 1.0	0.17	−2.2	−6.3; 1.8	0.28
Diastolic blood pressure (mmHg)	−0.9	−3.8; 2.1	0.55	−1.6	−5.4; 2.1	0.38
*Women*						
BMI (kg/m^2^)	−0.7	−1.7; 0.2	0.14	−0.7	−1.7; 0.3	0.18
Waist circumference (cm)	0.7	−1.5; 2.9	0.53	1.1	−1.2; 3.3	0.35
Body roundness index	0.1	−0.2; 0.3	0.60	0.1	−0.2; 0.3	0.68
Systolic blood pressure (mmHg)	−1.6	−4.3; 1.2	0.26	−0.3	−3.7; 3.0	0.85
Diastolic blood pressure (mmHg)	−0.5	−2.8; 1.8	0.66	−0.7	−3.2; 3.5	0.67

^∗^Adjusted for age, physical activity (hours sport/week), alcohol consumption, current BMI mother, and current BMI father; ^∗∗^Unstandardized regression coefficients of ICSI versus controls; *P* values indicate if these are significantly different from 0 (no effect)

**Table 3:** Metabolic blood markers in ICSI-conceived and control men and women.

	Men			Women		
	ICSI *N* = 56	Control *N* = 53	*P* value	ICSI *N* = 70	Control *N* = 80	*P* value
Total cholesterol (mg/dl)						
Mean (SD)	153.9 (24.8)	153.7 (23.5)	0.97	184.8 (33.9)	175.2 (31.3)	0.07
HDL cholesterol (mg/dl)						
Mean (SD)	47.9 (9.0)	52.4 (11.0)	0.02	65.8 (17.2)	64.2 (16.7)	0.55
LDL cholesterol (mg/dl)						
Mean (SD)	90.6 (20.2)	86.3 (22.8)	0.31	99.8 (28.1)	93.1 (27.2)	0.21
Triglycerides (mg/dl)						
Mean (SD)	82.7 (38.3)	75.8 (28.3)	0.28	103.5 (47.0)	93.3 (36.4)	0.14
Back-transformed mean (95% CI)	74.8 (66.2; 84.5)	71.1 (64.4; 78.5)	0.52	93.6 (84.7; 103.5)	87.2 (80.4; 94.6)	021
Glucose (mg/dl)						
Mean (SD)	82.4 (6.8)	84.1 (6.1)	0.18	79.5 (5.6)	81.7 (6.7)	0.03
Insulin (pmol/l)						
Mean (SD)	63.7 (42.0)	53.7 (24.9)	0.13	66.7 (28.2)	67.2 (24.9)	0.91
Back-transformed mean (95% CI)	54.3 (47.0; 62.8)	49.1 (43.8; 55.1)	0.28	61.7 (56.1; 67.9)	63.1 (58.3; 68.3)	0.72
HOMA-IR						
Mean (SD)	1.8 (1.2)	1.6 (0.8)	0.19	1.9 (0.8)	1.9 (0.8)	0.64
Back-transformed mean (95% CI)	1.6 (1.4; 1.8)	1.5 (1.3; 1.6)	0.40	1.7 (1.6; 1.9)	1.8 (1.7; 1.9)	0.44

*P* values for the comparison of ICSI versus controls with a *t*-test.

**Table 4:** Risk factors of the metabolic syndrome^∗^ in ICSI-conceived and control men and women.

	Men			Women		
	ICSI *N* = 56	Control *N* = 53	*P* value	ICSI *N* = 70	Control *N* = 80	*P* value
Waist circumference > 102 cm for men	0 (−)	1 (2%)	0.48			
Waist circumference > 88 cm for women				0 (−)	1 (1%)	1.0
Systolic BP ≥ 130 mmHg or diastolic BP > 85 mmHg	12 (21%)	14 (26%)	0.65	6 (9%)	5 (6%)	0.75
Triglycerides ≥ 150 mg/dl	3 (5%)	1 (2%)	0.62	11 (16%)	9 (11%)	0.47
HDL cholesterol < 40 mg/dl for men	11 (20%)	3 (6%)	0.04			
HDL cholesterol < 50 mg/dl for women				11 (16%)	16 (20%)	0.53
Glucose ≥ 100 mg/dl	0 (−)	0 (−)		0 (−)	0 (−)	
Metabolic syndrome (3 or more components)	0 (−)	0 (−)		0 (−)	1 (1%)	
Two cardiometabolic risk factors	2 (4%)	2 (4%)		3 (4%)	3 (4%)	

^∗^Number (%); *P* values for the comparison of ICSI versus controls with the Fisher's exact test.
